# Androgen receptor activation promotes tumor progression in canine and human triple negative breast cancer cell lines

**DOI:** 10.3389/fvets.2025.1677830

**Published:** 2025-09-23

**Authors:** Sara Caceres, Belen Crespo, María Herrera, Miriam de la Puente, Cristina Diaz Del Arco, Angela Alonso-Diez, Maria Jose Illera, Juan Carlos Illera

**Affiliations:** ^1^Department of Animal Physiology, School of Veterinary Medicine, University Complutense of Madrid, Madrid, Spain; ^2^Department of Obstetrics and Gynecology, Instituto de Salud de la Mujer, Instituto de Investigación Sanitaria del Hospital Clínico San Carlos (IsISSC), Madrid, Spain; ^3^Department of Public and Maternal Child Health, School of Medicine, University Complutense of Madrid, Madrid, Spain; ^4^Department of Surgical Pathology, Hospital Clínico San Carlos, Madrid, Spain; ^5^Department of Animal Medicine, Surgery and Pathology, School of Veterinary Medicine, University Complutense of Madrid, Madrid, Spain

**Keywords:** triple negative breast cancer, androgen receptor, ailanthone, steroid hormones, canine model

## Abstract

**Introduction:**

Triple negative breast cancer (TNBC) is an aggressive type of breast cancer that lack of expression of hormonal receptors and HER-2 that limits the approach of effective therapies. Currently, the expression of the androgen receptor (AR), and its prognostic potential are being explored in these tumors. Therefore, this study aimed to determine the mechanisms of action of AR in TNBC and the potential of AR antagonists as treatment in canine (IPC-366) and human (SUM149) TNBC cell lines.

**Methods:**

To achieve this, AR silencing assays were performed to determine evaluate the changes in AR signaling and the role of AR in cellular processes. Also, the effect of different AR-antagonists was evaluated on both cell lines.

**Results:**

The findings showed that AR promotes tumor progression by upregulating EGFR expression, which drives cell proliferation through the MAPK and PI3K signaling pathways. Additionally, AR downregulated Src expression, preventing the antiproliferative effects of ERβ, thus ensuring cancer cell survival. The study found that AR activation in TNBC is largely dependent on hormonal signals, highlighting the importance of the balance between androgen and estrogen levels.

**Discussion:**

Finally, results revealed that ailanthone acted as a potent AR antagonist, effectively blocking AR and Src expression in both canine and human cell lines, reducing significantly cell proliferation. The study concludes that AR and the tumor’s hormonal environment are critical for TNBC progression and that ailanthone could be a beneficial treatment for both human and canine TNBC.

## Background

Triple negative breast cancer (TNBC) is defined as a heterogeneous group of breast cancers characterized by the absence of estrogen receptor (ER), progesterone receptor (PR) and human epidermal factor 2 (HER2) expression. Due to the lack of specific targets, treatment options are limited, remaining the cytotoxic chemotherapy as the standard therapeutic option obtaining poor outcomes (1, 2). The natural heterogeneity of this disease turns TNBC into the most aggressive breast cancer subtype with high recurrence and poor prognosis that can be divided into six different subclasses of TNBC based on molecular analysis (2–4). One of the subtypes that Lehman’s classification contemplated was the luminal-androgen receptor (LAR), characterized by presenting a high expression of genes associated with the androgen receptor (AR) (3, 4).

AR expression in breast cancer is approximately 70–80% (5) and varies from 20 to 50% in TNBC patients regardless of the subtype (6), and its positivity is associated with favorable clinical features in hormone positive-receptor tumors (7, 8) and in TNBC (5). AR is a steroid receptor that belongs to the Type I class of nuclear hormone transcription factors. Its inactive form is located in the cytoplasm bind to heat shock proteins (HSP) and it is activated when androgens bind to AR’s binding domain (LBD) and displacing the HSP bound. This results in a conformational change that promotes the translocation of AR to the nucleus, binds to androgen response elements (ARE) activating gene transcription and influencing functions such as cell growth, migration, and apoptosis (9). Indeed, AR can be activated by non-genomic actions that involve the activation of different signaling pathways including phosphoinositide 3-kinase / protein kinase B (PI3K/Akt) and mitogen-activated protein kinases (MAPK) pathways (9, 10), and also, can be activated by its interaction with other receptors such as the epidermal growth factor receptor (EGFR) (11, 12). Particularly, AR positivity in TNBC has been related to estrogen receptor beta (ERβ) expression (13, 14). ERβ exerts its action by upregulating the protein phosphatase and tensin homolog (PTEN) and consequently decreasing the activation of the PI3K/Akt pathway and downregulating AR expression (1, 13, 14).

Scarce data exists regarding the function of the androgen receptor (AR) and the role of androgens in triple-negative breast cancer (TNBC). While several studies propose that AR drives cell proliferation, others suggest an antiproliferative role (15). This scenario is further complicated by the existence of up to 22 splice variants of AR. These variants, which have been described in prostate tumors, have been linked to resistance to AR-targeted therapies. Whereas the variant 7 (ARV7) has been found to be the most clinically relevant variant in prostate cancer, its role in breast cancer remains unraveled (16). However, it has been observed that ARV7 is also the predominant AR variant expressed in breast specimens and its presence is associated with poor clinical outcomes and resistance to endocrine therapies (7, 17, 18).

Based on these findings, AR has emerged as a potential target for TNBC treatment. One of the hallmark therapies in prostate cancer is androgen depletion therapy and thanks to advances in this type of cancer, more potent and promising new therapies that block AR signaling continue to be developed (19). The first-generation of AR antagonists such as bicalutamide or nilutamide, exerts its action blocking the AR activation. These compounds have been explored in TNBC demonstrating to be safe and improve patient survival (20). Those compounds were developed for targeting AR-LBD domains, and it are often associated with a resistance acquisition in prostate cancer, denoting the emerge on developing next-generation compounds that impact to other AR domains or mutants (20). In this regard, two other promising molecules are VPC-13566 and Ailanthone. While VPC-13566 targets the AR binding function 3 (BF·3) inhibiting AR transcriptional activity, ailanthone inhibits transcriptional activity of full-length and AR splicing variants, presenting both excellent results as anticancer drugs in prostate cancer (21–23).

Since all these therapies are widely studied in prostate cancer, breast cancer remains limited, in part, due to the complexity of AR signaling in these tumors (5). It has been shown that canine and human breast cancer share biological and molecular characteristics denoting that the canine model can be a useful tool for comparative (24). Research on canine model has provided results that can be applied to human research (25, 26). Therefore, advances in research into new therapies using canine and human research models may offer advantages for both species.

Overall, the aim of this study is to identify AR as a promising biomarker for the diagnosis and treatment of TNBC. To this purpose, the mechanism of action of AR is evaluated in TNBC cells that differ in the intensity of its expression, exploring the changes in AR signaling when cells are exposed to different AR antagonists.

## Methods

### Cell culture

Canine IPC-366 and human SUM149 cell lines were chosen for this study since both are inflammatory TNBC cell lines that shared biological and histopathological characteristics (24).

IPC-366 cell line was obtained from the Department of Physiology, School of Veterinary Medicine (University Complutense of Madrid, Spain). This cell line is a canine inflammatory mammary cancer cell line with high AR-positivity (25). It was cultured in Dulbecco’s modified Eagle medium nutrient mixture F-12 Ham (DMEM/F12) (Sigma-Aldrich, Missouri, USA), supplemented with 5% charcoal-stripped fetal bovine serum (CH-FBS), 1% penicillin–streptomycin solution, and 1% L-glutamine (Sigma-Aldrich, Missouri, USA). On the other hand, SUM149 cell line, a human inflammatory breast cancer cell line with a low AR-positivity (25), was purchased from Asterand, Plc. (Detroit, Michigan, USA). It was cultured in Nutrient mixture F-12 HAM medium (Sigma-Aldrich, Missouri, USA) supplemented with 5% CH-FBS, 1 μg/mL hydrocortisone, 5 μg/mL insulin, and 1% penicillin–streptomycin solution (Sigma-Aldrich, Missouri, USA).

The cells were cultured in 25-cm^2^ culture flasks and maintained at 37 °C in a humidified 5% CO_2_ atmosphere. Cell cultures were monitored daily by phase-contrast microscope (Optika XDS-2 Inverted Microscope, Euromicroscopes, S. L., Barcelona, Spain) to determine cell viability and growth.

### Treatments

The AR antagonists used in this study were as follows: nilutamide and bicalutamide (Sigma-Aldrich, Missouri, USA), non-steroidal antiandrogens that block AR and are widely used for prostate cancer treatment (23); and VPC-13566 and ailanthone (Sigma-Aldrich, Missouri, USA), that induce protein degradation of full-length and splicing variant AR proteins (23). Treatments were dissolved in dimethyl sulphoxide (DMSO) at a stock concentration of 10 mM and stored at −20 °C until use.

### Sensitivity assays

In order to determine the half maximal effective concentration (EC-50) of each AR antagonist *in vitro*, a sensitivity assay was performed (26). Briefly, IPC-366 and SUM149 cells were seeded at a density of 10^3^ cells per well in 96-well polystyrene plates (Corning Incorporated, New York, USA) in culture media and administered 5-fold serial dilutions of each compound starting at a dose 10 mM to 64 nM. Control cells were treated with DMSO at a final concentration <0.1%. Then, cells were incubated for 72 h at 37 °C in a humidified 5% carbon dioxide atmosphere. Finally, bromide of 3-(4,5-dimetiltiazol-2-ilo)-2,5-difeniltetrazol (MTT) (Sigma-Aldrich, Missouri, USA) was added in all wells and the absorbances were measured at a wavelength of 568 nm with an automatic plate reader (ThermoFisher Scientific, Massachusetts, USA). Sensitivity curves were processed with GraphPad Prism 6.01 software to obtain the EC-50 values of each compound. The assay was carried out in duplicate.

Sensitivity results revealed that IPC-366 and SUM149 were sensitive to all treatments at a concentration of 1 μM, thus, *in vitro* cell viability and migration assays were performed at a concentration of 1 μM for each compound.

### Cell viability and migration assays

AR antagonists were administered to IPC-366 and SUM149 cells to evaluate cell viability and migration characteristics. For cell viability assay, cells were cultured in 96-well polystyrene plates at a density of 10^4^ cells per well in culture media and were administered with 1 μM of nilutamide, bicalutamide, VPC-13566, and ailanthone. Cells treated with DMSO were considered control group. Cells were maintained for 24 h at 37 °C in a humidified 5% CO2 atmosphere. Two experiments were carried out by duplicates. Then, MTT was added to all wells and the absorbances were measured at a wavelength of 568 nm with an automatic plate reader. Results were expressed as percentage of viable cells respect to control.

Likewise, for migration assays 10^5^ IPC-366 and SUM149 cells per well were cultured in 24-well polystyrene plates (Corning Incorporated, New York, USA). When the cells reached a confluence of 90%, a wound was performed in the middle of the well and the compounds were added to each well. Cells were incubated at 37 °C in a humidified 5% carbon dioxide atmosphere for 24 h. Two experiments were carried out by duplicates. Images for each well were taken with a phase-contrast microscopy (Optika XDS-2 Inverted Microscope, Euromicroscopes, S. L., Spain) at the time of performing the wound (zero hours) and 24 h after. Besides, culture media was collected at 24 h for hormonal analysis.

Data were processed by ImageJ MRI-Wound Healing Tool software 1.53e version, comparing the wound width at zero and 24 h of the different treatments with respect to the control. Measures were obtained in pixels and represented as a percentage of wound closure with respect to the control group.

### siAR transfections

IPC-366 cells were cultured in 12-well plates. When cells reached 80–85% confluence, 0.1 μM siRNA against AR (siAR) (s1539; s1540, Thermo Fisher, Scientific, Massachusetts, USA) and 0.1 μM siRNA negative and positive controls (siCNT) (4,390,843; 4390849, Thermo Fisher, Scientific, Massachusetts, USA). The transfection was performed using Lipofectamine® RNAiMAX (ThermoFisher, Scientific, Massachusetts, USA) according to manufacturer’s instructions. Cells were incubated for 24 h at 37 °C and subsequently media was replaced with supplemented media for 24 h. Then, media from all wells was collected and stored at −20 °C for hormonal determinations. siCNT and siAR cells were washed and used both for cell viability and tumor growth assays. A part of the control and siAR, cells were used to obtain protein extracts for transfection evaluation by Western blot analysis. The assay was carried out in duplicate.

### DHT and E2 administration

Once the transfection was complete, IPC-366 control and siAR cells were harvested and cultured in 96-well polystyrene plates at a density of 10^4^ cells per well in fresh culture media or supplemented with 100 nM of dihydrotestosterone (DHT) or 17β-estradiol (E2) (Steraloids Inc. Newport, R. I) and maintained at 37 °C in a humidified 5% CO2 atmosphere for 24 h. The selection of the dose was based on previous studies (27). Then, culture media from control and siAR cells administered with DHT and E2 were collected and MTT was added to all wells. The absorbances were measured at a wavelength of 568 nm with an automatic plate reader and results were expressed as percentage of viable cells respect to control or siAR. Two experiments were carried out by duplicates.

### Tumor growth assay

A total of twenty 6-to-8 weeks old female immunocompromised Balb/SCID mice obtained from Janvier Labs (Madrid, Spain), were maintained and acclimatized for 7 days in the Animal Facility (Department of Animal Physiology, School of Veterinary Medicine, University Complutense of Madrid). The mice were housed in polycarbonate cages (three animals per cage) in a room with controlled environmental conditions (temperature: 23 ± 2 °C; relative humidity: 50 ± 10%; 10–15 air changes per hour; and a light:dark cycle of 12:12 h). Soy-free pellet food (Dyets, Inc., Bethlehem, Pennsylvania, USA) and water, previously sterilized, were provided *ad libitum*. The required sample size needed to simultaneously compare the normal means of the groups was determined using the sample size determination module of the statistical package Statgraphics Centurion XVI (Statpoint Technologies, Inc., Warrenton, Virginia, USA). The experimental protocols were approved by the Institutional Animal Care and Use Committee of the Complutense University of Madrid, Spain (number: Proex 176/19). All procedures were performed in accordance with the Guide for the Care and Use of Laboratory Animals and conformed with the relevant EU Directive and the ARRIVE guidelines 2.0.

Animals were anesthetized prior to all procedures with isoflurane (IsoVet) at 4% for induction and 1.5% to maintain sedation, supplied in a fresh gas flow rate of 0.5 L oxygen/min, and were observed until fully recovery. A suspension of 10^6^ siCNT and siAR IPC-366 cells diluted in PBS were inoculated in the mammary fat pad of 10 female mice, respectively. The mice were inspected twice weekly for the development of tumors until a volume of 0.5 cm3 was reached. Then, mice were monitored by palpation and tumors were measured using calipers every 2 days for 15 days. Tumor volume was estimated using the following formula: volume = ((length) x (width)^2)/2 (28). When tumors reached a volume of 1.5 cm3 (endpoint), mice were sacrificed with a lethal dose of isoflurane. Prior to sacrifice, blood samples were obtained by intracardiac puncture. At necropsy tumors were harvested and divided into two fragments: one for histological analysis and the other for hormonal analysis.

The tumor fragment for hormonal analysis was homogenized in PBS, centrifuged at 1200 *xg* for 20 min at 4 °C, and frozen at −20 °C until hormonal analysis. Blood samples were also centrifuged at 1200 *xg* for 20 min at 4 °C and serum was separated and stored at −20 °C until hormonal analysis.

### Steroid hormone determinations

Progesterone (P4), androstenedione (A4), estrone sulfate (E1), E2, and testosterone (T) levels were determined in culture media from *in vitro* assays and tumor and serum samples using an enzyme-immunoassay (EIA) previously validated (29, 30). The antibodies used for this technique were developed in the Department of Physiology (UCM, Spain). Samples were run in duplicates in two different replicates.

Dihydroepiandrostenedione (DHEA) and DHT determinations were performed using a commercially available EIA kit (Demeditec, Germany) according to the manufacturer’s instructions.

Hormone concentrations were calculated using a software developed for this technique (ELISA AIS, Eurogenetics, Belgium). A standard dose–response curve was constructed by plotting the binding percentage (B/BO × 100) against each standard concentration of steroid hormone. All hormone concentrations were expressed in ng/ml for culture medium and serum, and in ng/g for tumor homogenates; DHT culture media, serum and tumor homogenate hormone concentrations were expressed in pg./ml and pg./g, respectively. DHT/E2 ratios were calculated as DHT concentrations divided by E2 concentrations. Ratios were expressed as mean percentages.

### Western blot analysis

Expression of proteins related to AR signaling was evaluated in siCNT, siAR, and IPC-366 and SUM149 treated cells. Expression of protein levels of AR, ARV7, phospo-AR (pAR), ERβ, EGFR, signal transducer and activator of transcription 3 (STAT3), Ras, Src, and extracellular response kinases 1 and 2 (ERK1/2) was assayed by western blot techniques. The total proteins from IPC-366 and SUM149 control, siAR, and treated cells, were extracted in RIPA buffer with protease and phosphate inhibitors (ThermoFisher Scientific, Massachusetts, USA). Protein concentrations were determined with a BCA Protein Assay Kit (ThermoFisher Scientific, Massachusetts, USA) following manufacturer’s instructions. Then, proteins were denaturalized adding LDS sample buffer that contains lithium dodecyl sulfate (ThermoFisher Scientific, Massachusetts, USA) to 40 μg of protein extracts and boiled at 70 °C for 10 min before loading. Samples were separated on NuPAGE Bis-Tris 4–12% gels (ThermoFisher Scientific, Massachusetts, USA) and transferred to iBlot 2 transfer stacks nitrocellulose membranes (ThermoFisher Scientific, Massachusetts, USA). Subsequently, membranes were blocked with 5% nonfat dried milk (Panreac Applichem, Barcelona, Spain) for 1 h at room temperature and probed overnight at 4 °C with the correspondence dilution of primary antibodies. Anti-AR (PA1-37079; 1/500); anti-p-AR (PA5-106115;1/1000); anti-ERβ (51–7,700; 1/500); anti-EGFR (PA1-1110; 1/1000); Anti-STAT3 (MA1-13042; 1/2000); Anti-Ras (PA5-87037; 1/1000); Anti-Src (44–655; 1/1000); Anti-Akt (PA5-104548; 1/1000); Anti-ERK1/2 (MA5-15134; 1/1000) (ThermoFisher Scientific, Massachusetts, USA); ARV7 (68,492; 1/500) (Cell Signaling Technology, Danvers, MA, USA), and GADPH (used as loading control) (HRP-60004; 1/10,000) (Proteintech, Planegg-Martinsried Germany), were used as primary antibodies. After washing the membranes with Tris-buffered saline plus Tween20, the secondary antibody, Goat anti-Rabbit IgG (H + L) HRP (32,460; 1/1000) or Goat anti-Mouse IgG (H + L) HRP (32,430; 1/1000), was added to the membranes and incubated for 1 h at room temperature. The protein expression was observed by chemiluminescence with Supersignal West Pico PLUS (ThermoFisher Scientific, Massachusetts, USA) and visualized with Alliance Q9 Atom imaging system (Uvitec, Cambridge, UK). Images processed and quantification were performed using the Alliance Q9 software.

### Statistics

Data was analyzed by IBM SPSS Statistics 28.0 software. The Shapiro-Wilks test was used to assess the goodness-of-fit distribution of the data. For the data that were normally distributed, a one-way analysis of variance (ANOVA) followed by a Bonferroni test, was used to establish significant differences between control and experimental groups in terms of cell viability and migration assays, culture hormonal analysis, tumor growth, and western blot quantification analysis. Besides, for samples that were not normally distributed, a non-parametric Mann–Whitney U test was performed to establish significant differences between controls and treatments in serum and homogenate hormone determinations. In all statistical comparisons, *p-*values of *p* < 0.05 were considered statistically significant.

## Results

### AR expression and hormone secretion in IPC-366 and SUM149 cells

**Figure 1:**
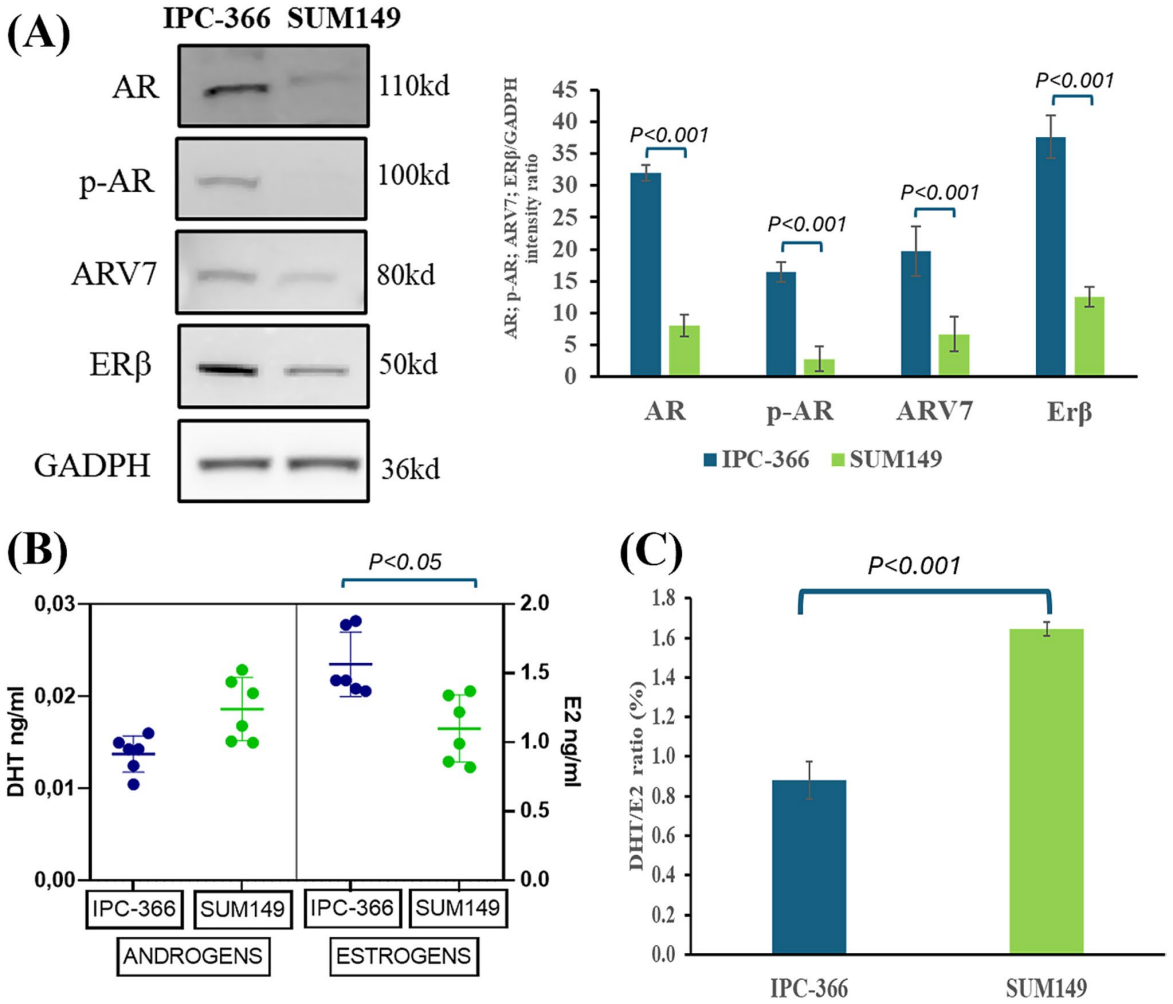
**(A)** AR, p-AR, ARV7, and ERβ western blot analysis expression in IPC-366 and SUM149 cells. Graph represents AR, p-AR, ARV7, and ERβ relative quantification in both cell lines. **(B)** DHT (androgens) and E2 (estrogens) secreted by IPC-366 and SUM149 cells determined by EIA technique. **(C)** DHT/E2 ratio expressed as percentage calculated for IPC-366 and SUM149.

Results showed that IPC-366 and SUM149 cells expressed AR, p-AR, AR-V7, and ERβ, denoting significant differences (*p* < 0.05) in expression between IPC-366 and SUM149 cells, being higher in IPC-366 and weak in SUM149 (Figure 1A). Regarding androgens (DHT) and estrogens (E2) secretion, results revealed that both cell lines secreted more estrogen levels than androgens in *in vitro* conditions (Figure 1B). Indeed, significantly higher estrogen secretion (*p* < 0.05) was found in IPC-366 cells than in SUIM149 cells, while no significant differences were observed in androgen secretion, although SUM149 secreted higher androgen levels. These differences in estrogen and androgen levels contributed to find statistical differences in DHT/E2 ratio between both cell lines (Figure 1C). Results showed that SUM149 presented a significantly higher DHT/E2 ratio (*p* < 0.05) than IPC-366.

### Steroid hormone administration altered AR signaling in IPC-366 siAR cells

**Figure 2:**
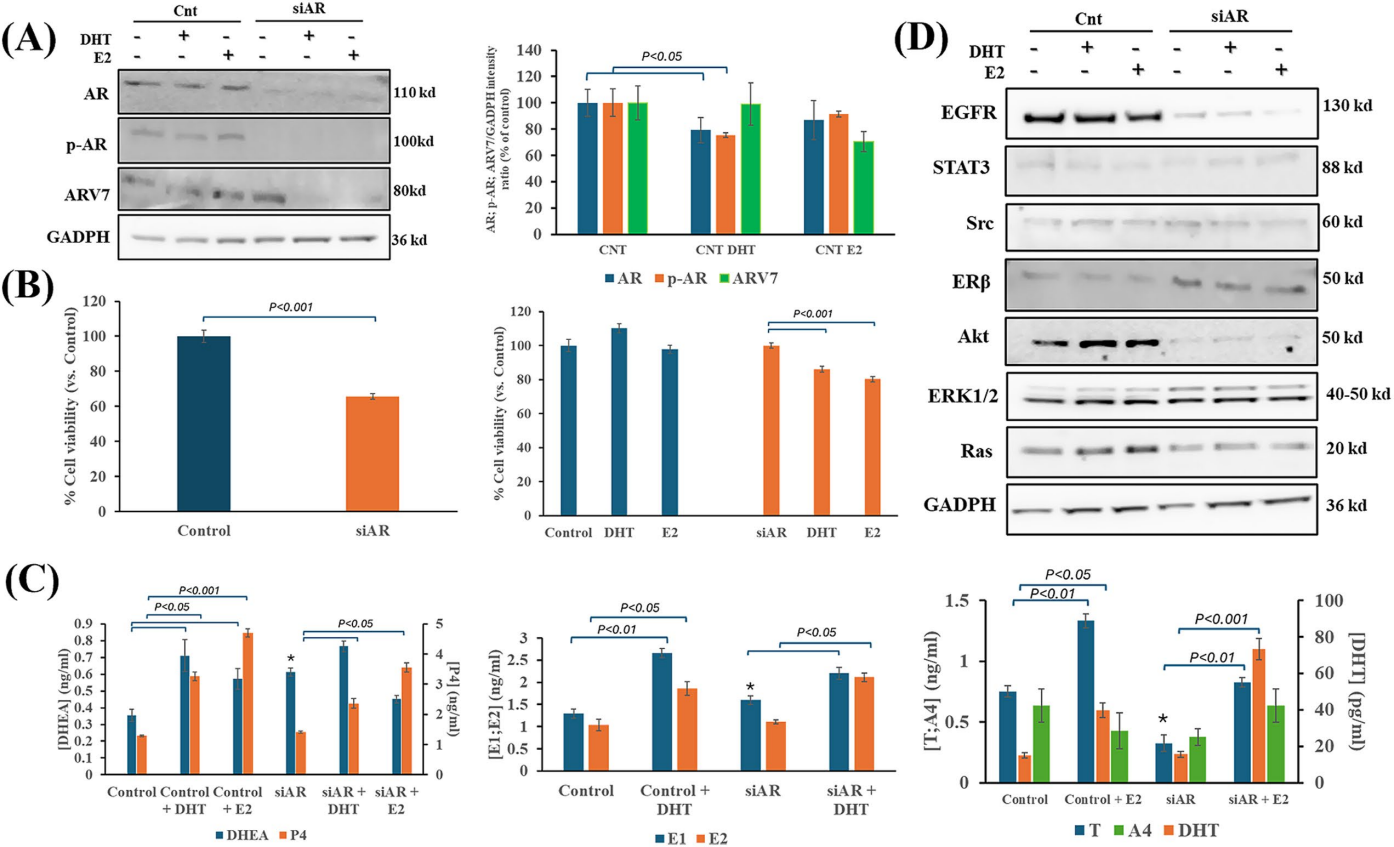
Effects of DHT and E2 administration on IPC-366 CNT and siAR cells. **(A)** AR silencing in IPC-366 cells. AR, p-AR and ARV7 expressions in IPC-366 control with and without E2 and DHT administration. Bars represent protein intensity respect to GADPH expression and data was represented as percentage respect to control (CNT) group. **(B)** Graphs represent percentage of cell viability of siCNT and siAR cells (left), and after DHT or E2 administration. **(C)** Steroid hormone concentrations secreted by siCNT and siAR cells with or without DHT and E2. Bars represent ± SD of DHEA, P4 (upper), E1, E2 (middle), T, A4, and DHT (lower) concentrations. *Denoted significant differences (*p* < 0.05) between siCNT and siAR. **(D)** EGFR, STAT3, Src, Erβ, Akt, ERK1/2 and Ras protein expression in control and siAR cells and with E2 or DHT administration.

The presence or absence of AR expression in IPC-366 cells and the changes in hormonal environment resulted in differences in cell viability, hormone secretion and protein expression. Silencing AR expression (siAR) resulted in inhibiting AR full length and p-AR expression but failed in silencing ARV7 expression (Figure 2A). The administration of DHT or E2 to siAR cells showed that ARV7 expression can be significantly reduced by hormone alterations. However, in IPC-366 control cells (CNT), the administration of DHT produced a significant reduction (*p* < 0.05) in AR and p-AR expressions, but not in ARV7 expression. On the other hand, siAR cells showed a significant reduction of cell viability compared to CNT cells (Figure 2B). Although CNT cells did not show significant changes in cell viability with E2 and DHT administration, siAR cells revealed a significant reduction (*p* < 0.05) in cell viability with these conditions. Results also indicated that CNT and siAR cells differed in steroid hormone secretion (Figure 2C). E1 and DHEA levels were significantly increased (*p* < 0.05) in siAR cells, while T levels were significantly decreased (*p* < 0.05) with respect to CNT cells. E2 administration to CNT and siAR cells, resulted in a significant increase (*p* < 0.05) in P4, DHEA and androgen (T and DHT) concentrations, while DHT administration produced a significant increase (*p* < 0.05) in P4, DHEA and estrogen (E1 and E2) concentrations.

siAR cells also showed differences in expression of proteins related to AR signaling compared to CNT cells (Figure 2D; Supplementary Figure S1). EGFR and Akt were significantly reduced (*p* < 0.05) in siAR cells, while ERβ, Src and ERK1 expression were significantly increased (*p* < 0.05) in siAR cells compared to CNT cells. Indeed, E2 and DHT administration to CNT cells produced a significant reduction (*p* < 0.05) in STAT3 expression, and a significant increase (*p* < 0.05) in Ras expression. However, in siAR cells, when E2 and DHT were administered a significant reduction (*p* < 0.05) in Src, ERβ and ERK1 expression was observed.

### Silencing AR expression reduced tumor progression in IPC-366 cells

**Figure 3:**
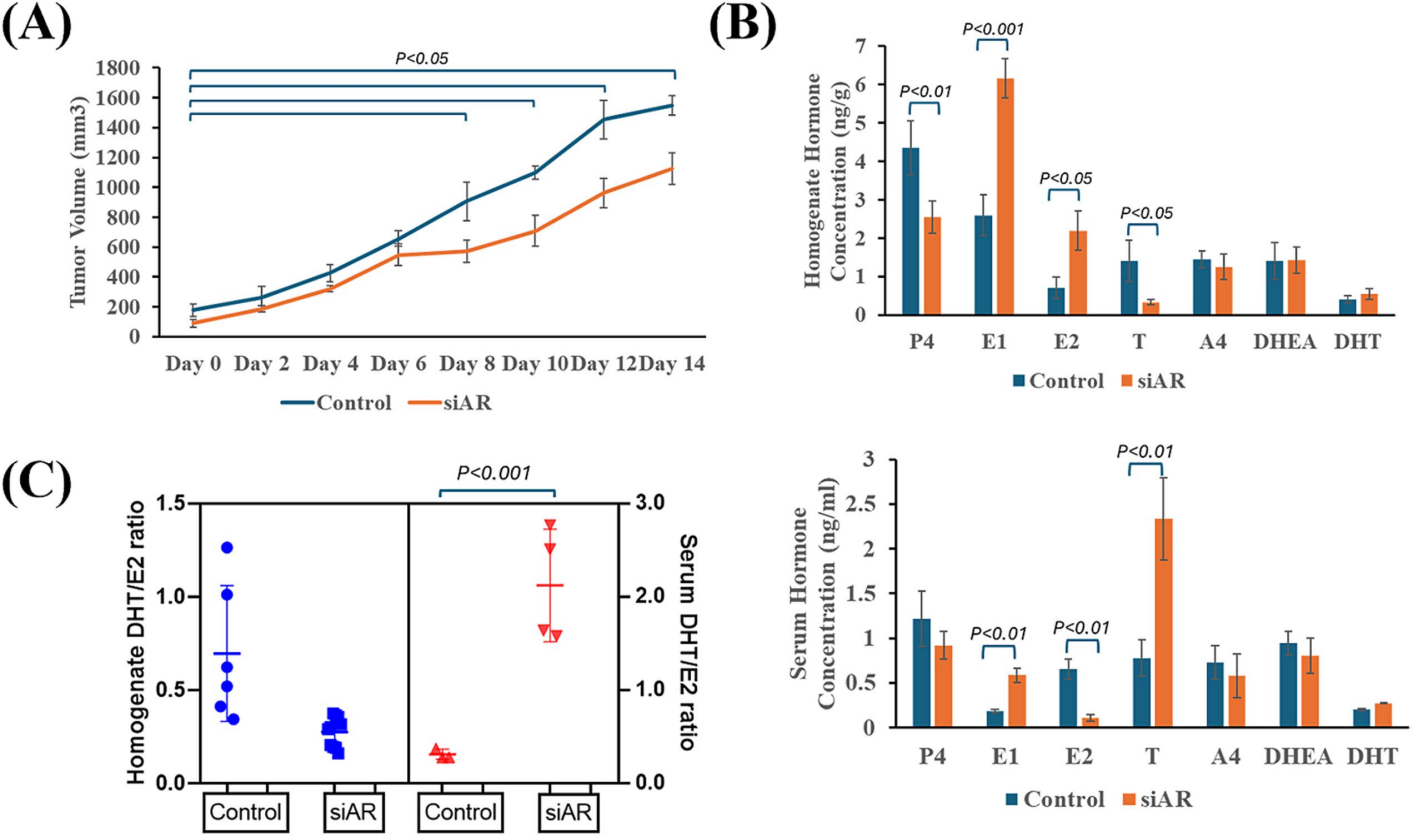
IPC-366 CNT and siAR in vivo tumor growth characteristics. **(A)** Graph represents tumor growth progression of IPC-366 CNT and siAR cells inoculated on SCID mice. **(B)** Bars represent steroid hormone determinations on tumor homogenates (upper) and serum samples (lower). **(C)** DHT/E2 ratio calculated in serum and tumor homogenate samples from CNT and siAR mice.

CNT and siAR cells were inoculated in female SCID mice in order to compare tumor progression and hormone secretion patterns. The results showed that in IPC-366 siAR mice, tumor progression was significantly slower (*p* < 0.05) than control mice from day 8 of tumor onset (Figure 3A).

Indeed, tumor homogenate and serum hormone concentrations differed from control and siAR tumors (Figure 3B). In siAR tumors, P4 and T concentrations were significantly reduced (*p* < 0.05) compared to control group, although estrogens (E1 and E2) were significantly higher (*p* < 0.05). In serum hormone levels, siAR mice showed a significant increase (*p* < 0.05) in E1 and T levels respect to control group, while E2 levels significantly decreased (*p* < 0.05). These hormonal differences were also notable in the calculated DHT/E2 ratio (Figure 3C). Results revealed that siAR tumor homogenates presented a lower DHT/E2 ratio than control tumors, but a significantly (*p* < 0.001) higher DHT/E2 ratio was found in serum siAR samples respect with control.

### IPC-366 and SUM149 cells were sensitive to AR antagonists

**Figure 4:**
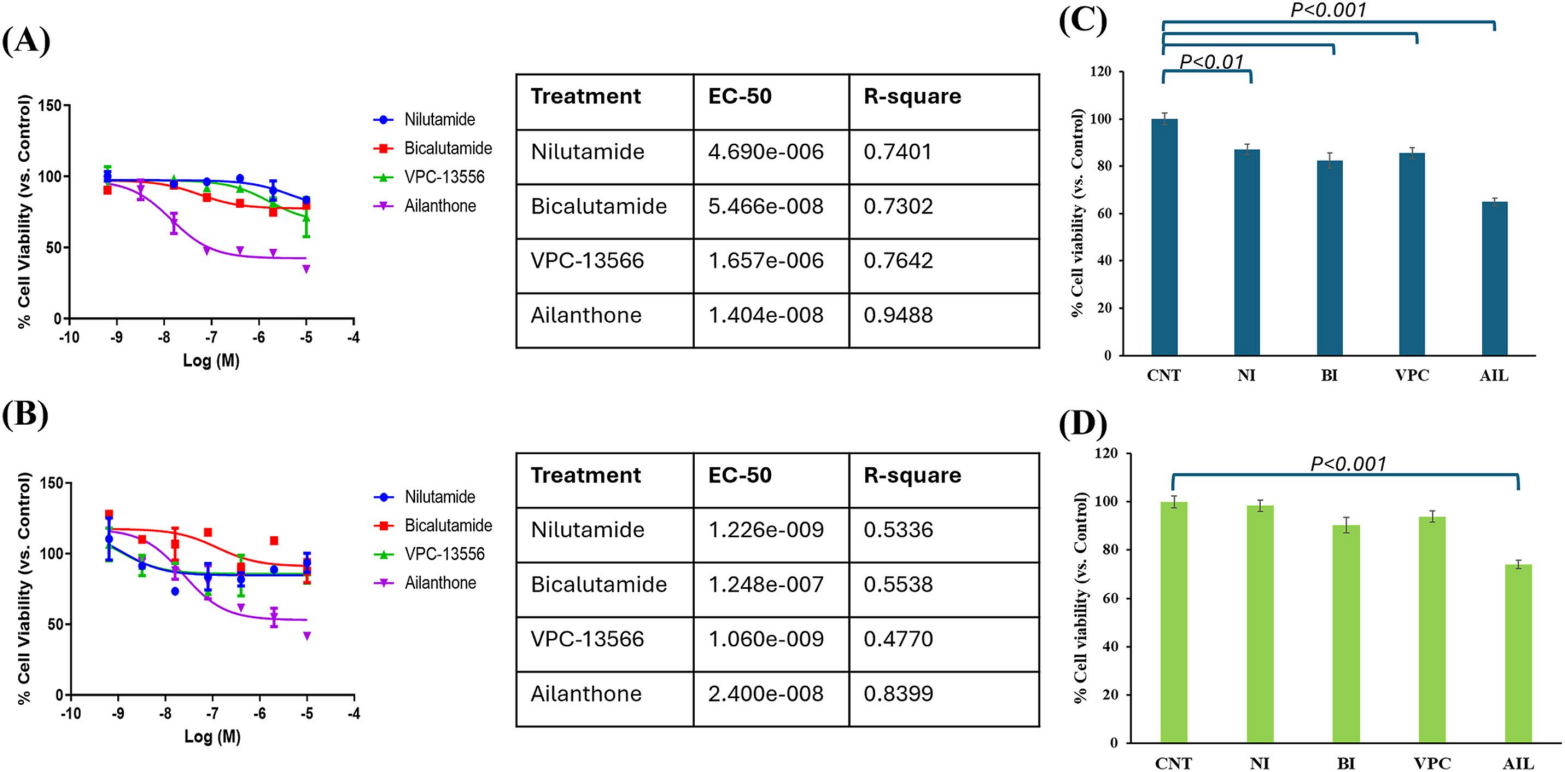
Sensitivity results from **(A)** IPC-366 and **(B)** SUM149 were carried out. Graphs represent percentage of cell viability respect to Control (CNT) in cells treated with nilutamide (NI), bicalutamide (BI), VPC-13566 (VPC), and ailanthone (AIL). The tables indicated the EC-50 values for each treatment and cell line. Cell viability results from **(C)** IPC-366 and **(D)** SUM149 were also performed. Bars represent percentage of cell viability with the administration of the compounds with respect to control group.

Considering that AR could be a good therapeutic target for TNBC cell lines, we explored the effect of different AR antagonists on cell lines with high and low AR expression (IPC-366 and SUM149, respectively). Sensitivity results from IPC-366 and SUM149 revealed that both cell lines were sensitive to all treatments analyzed (nilutamide, bicalutamide, VPC-13566 and, ailanthone), being SUM149 more sensitive than IPC-366 (Figures 4A,B). Particularly, ailanthone was the treatment that achieved better sensitivity results in both cell lines. These results were in line with those found in cell viability assay (Figures 4C,D). The viability of IPC-366 cells was significantly reduced (*p* < 0.05) in all treatment conditions compared to the control group. However, only the ailanthone treatment significantly reduced (*p* < 0.05) the cell viability of SUM149 cells compared to the control group.

### AR antagonists reduced cell migration in IPC-366 and SUM149 cell lines

**Figure 5:**
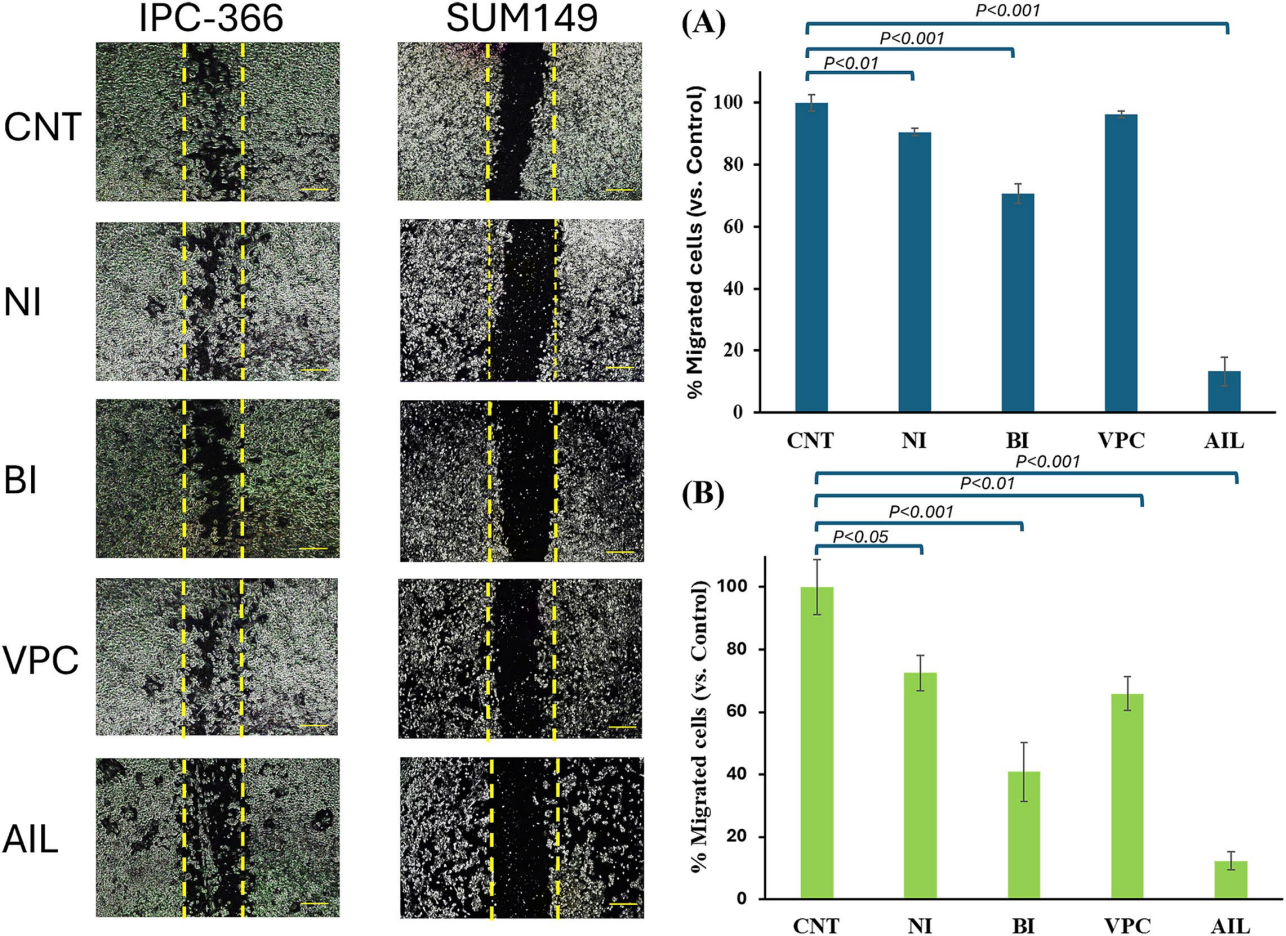
Migration assay. Images from wound closure and percentage of migrated cells of **(A)** IPC-366 and, **(B)** SUM149 cells untreated (CNT) and administered with nilutamide (NI), bicalutamide (BI), VPC-13566 (VPC), and ailanthone (AIL). Images were taken at a magnification of 10x (scale bar 1,000 μm) and processed with ImageJ MRI software. Bars represent percentage of migrated cells respect to control group.

In both cell lines, AR antagonists’ administration resulted in a reduction in the percentage of migrated cells. IPC-366 showed significant reductions (*p* < 0.05) in percentage of migrated cells under nilutamide, bicalutamide and ailanthone conditions. Also, administration of VPC-13566 showed a reduction in cell migration but not statistically significant (Figure 5A). Nevertheless, SUM149 cells showed significant reductions (*p* < 0.05) in cell migration with all treatments (Figure 5B). Interestingly, ailanthone reduced the percentage of migrated cells by approximately 87.24% ± 0.69 in both cell lines.

### AR antagonists altered steroid hormone secretion in IPC-366 and SUM149 cultured cells

**Figure 6:**
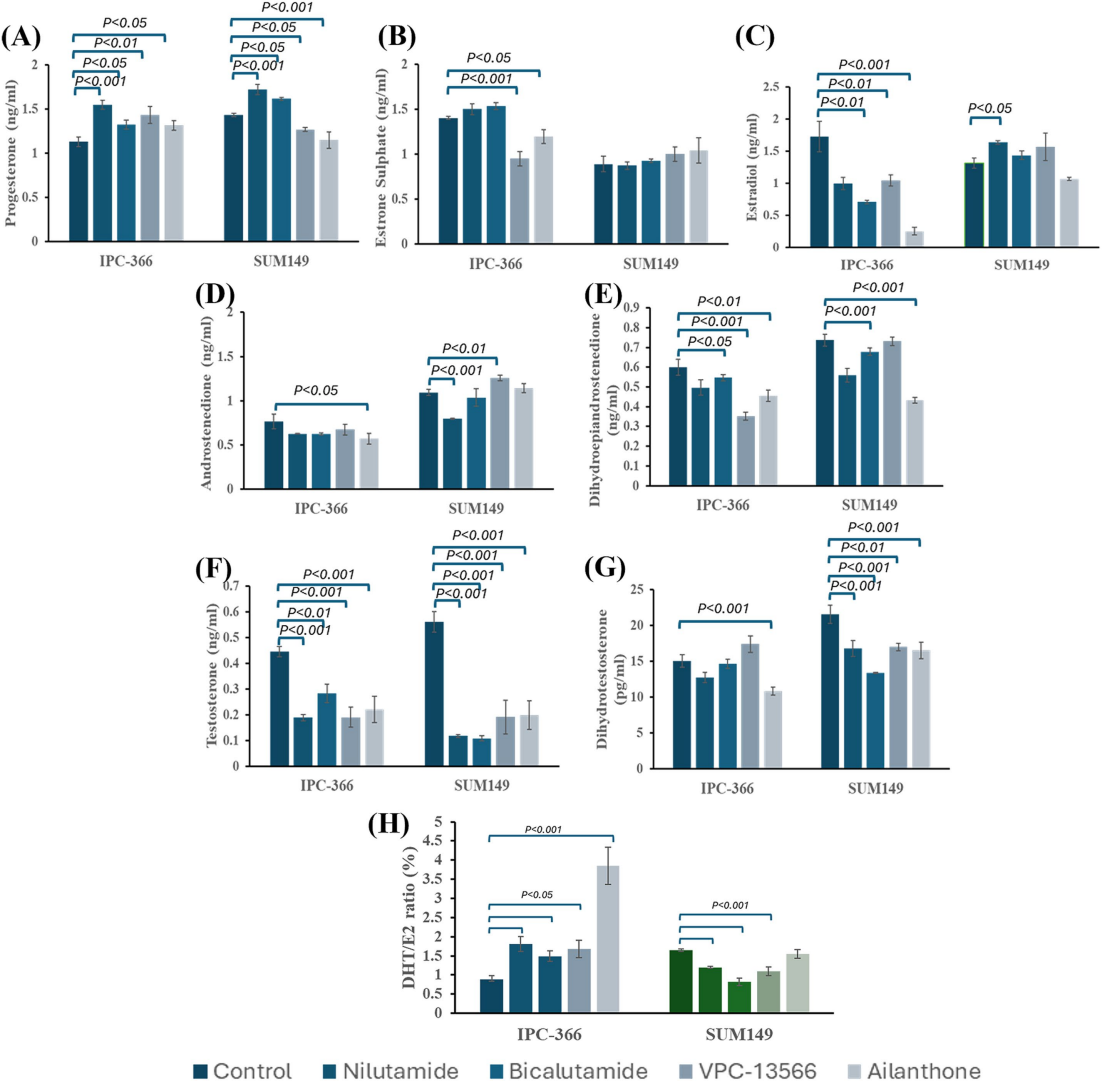
Steroid hormone concentrations of **(A)** P4; **(B)** E1; **(C)** E2; **(D)** A4; **(E)** DHEA; **(F)** T; and **(G)** DHT determined in culture media from IPC-366 and SUM149 cells untreated and treated with the different AR antagonists. **(H)** DHT/E2 ratio expressed as percentage.

In general, all AR antagonists administered to IPC-366 and SUM149 cells altered steroid hormone secretion by producing a decrease in hormone secreted levels (Figure 6). P4 levels showed a significant increase (*p* < 0.05) with all treatments in IPC-366 cells, while in SUM149, only nilutamide and bicalutamide significantly increased P4 secretion (*p* < 0.05) but VPC-13566 and ailanthone produced a significant decrease (*p* < 0.05) in P4 concentrations (Figure 6A).

Regarding estrogen levels, significant changes in E1 and E2 concentrations were found in IPC-366 cells, but no significant alterations were found in SUM149. IPC-366 showed a significant decrease (*p* < 0.05) in E2 levels with all treatments, and a significant decrease in E1 levels with VPC-13566 and ailanthone (Figures 6B,C).

Androgen levels were also decreased in both cell lines (Figures 6D–G). All treatments produced a significant drop (*p* < 0.05) in T levels in IPC-366 and SUM149 cells. However, DHT levels were only reduced in IPC-366 under ailanthone treatment, while in SUM149 all treatments reduced DHT levels significantly (*p* < 0.05). Similarly, A4 levels in IPC-366 were significantly decreased (*p* < 0.05) with ailanthone, while in SUM149 nilutamide decreased significantly (*p* < 0.05) A4 secreted levels, and VPC-13566 increased them significantly (*p* < 0.05). In addition, DHEA secreted levels were also reduced in IPC-366 and SUM149 cells, being this decreased significant (*p* < 0.05) under all treatments except for nilutamide and VPC-13566 in SUM149.

Besides, the differences found in androgen and estrogen secretion under treatment conditions, altered also DHT/E2 ratios respect to control (Figure 6H). In IPC-366, a significant increase in DHT/E2 ratio (*p* < 0.05) was found, while In SUM149 DHT/E2 ratio decreased significantly (*p* < 0.05), except for ailanthone treatment.

### AR antagonists reduced AR expression in IPC-366 and SUM149 cells

**Figure 7:**
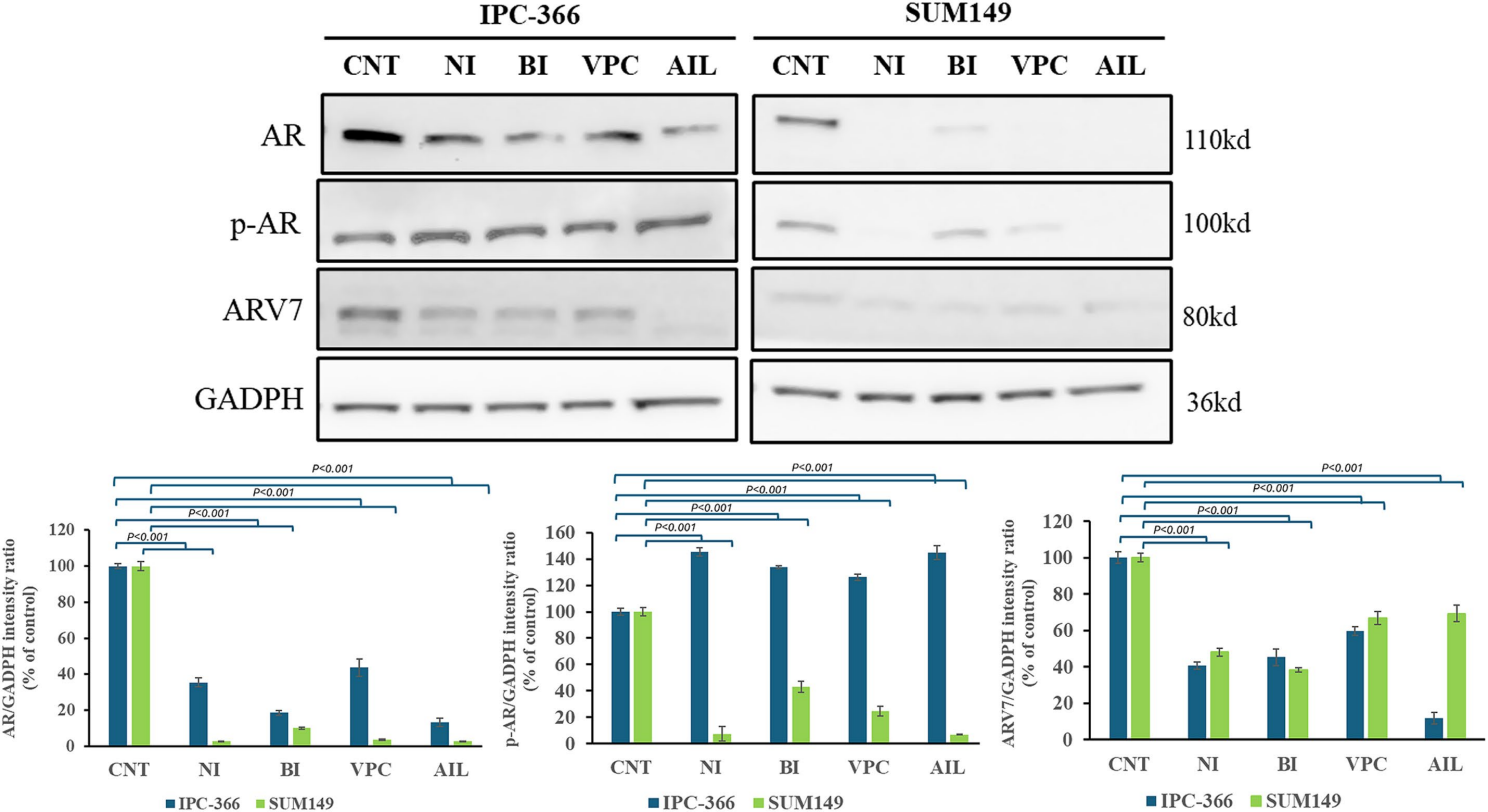
AR, p-AR and ARV7 expression in IPC-366 and SUM149 untreated cells (CNT) and treated with nilutamide (NI), bicalutamide (BI), VPC-13566 (VPC), and ailanthone (AIL). GADPH expression was determined as loading control. Bars represent AR, p-AR and ARV7 (from left to right) intensities respect to GADPH expression and data was represented as percentage respect to control (CNT) group.

The AR antagonists used in this study showed different effects in AR, p-AR and ARV7 expressions in IPC-366 and SUM149 cell lines (Figure 7). The results revealed that all treatments reduced significantly (*p* < 0.05) AR expression in both cell lines, but especially in SUM149. In this cell line, results also revealed a significant decrease (*p* < 0.05) in p-AR and ARV7 expression. However, in IPC-366 cells p-AR expression increased significantly (*p* < 0.05), but ARV7 expression showed a significant decrease (*p* < 0.05).

### AR signaling alterations with the administration of AR antagonists

**Figure 8:**
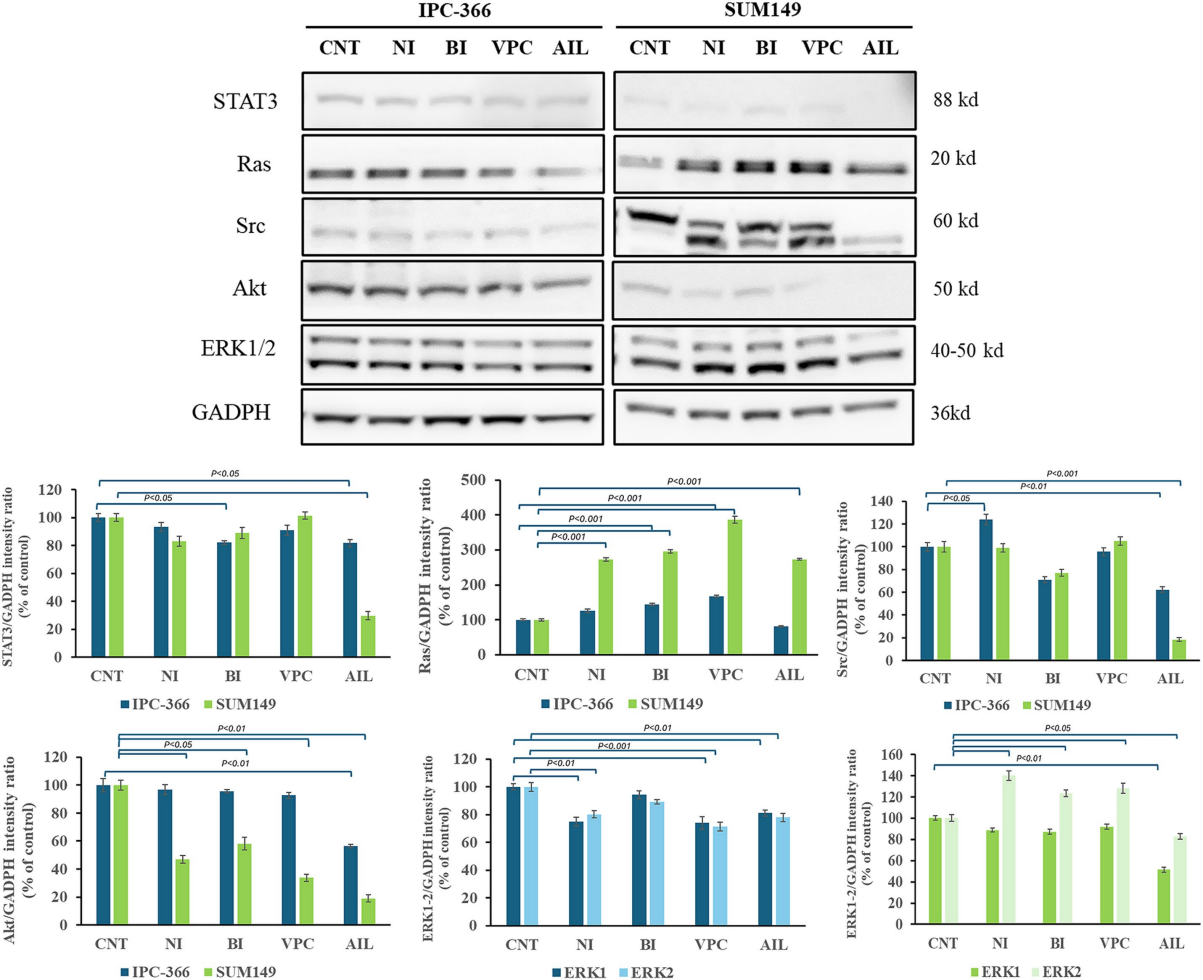
A STAT3, Ras, Src, Akt, and ERK1/2 protein expression in untreated and treated IPC-366 and SUM149 cells. GADPH expression was determined as loading control. Graphics of STAT3, Ras, Src, Akt, and ERK1/2 expression quantification. Bars represent protein intensity respect to GADPH, and data was represented as percentage respect to CNT group. ERK1/2 expression was represented in different graphics for IPC-366 (blue bars) and SUM149 (green bars).

The reduction of AR expressions by the administration of AR antagonists also produced alterations in the expression of AR signaling related proteins in both cell lines (Figure 8). Ras expression increased significantly (*p* < 0.01) in both cell lines with all treatments. However, STAT3 and Src expression in IPC-366 and SUM149 cells significantly decrease (*p* < 0.05; *p* < 0.01) with ailanthone administration. Similarly, Akt expression significantly decreased (*p* < 0.05) with all treatments in SUM149 cells, but in IPC-366 cells, only ailanthone significantly decreased (*p* < 0.01). In addition, ERK1/2 expression in IPC-366 cells was reduced with all treatments being significantly (*p* < 0.01) with the administration of nilutamide, VPC-13566, and ailanthone. However, in SUM149 cells, ERK1 expression did not showed any difference, but ERK2 expression significantly increased (*p* < 0.05) in cells treated with nilutamide, bicalutamide and VPC-13566.

### Crosstalk between AR and other related receptors

**Figure 9:**
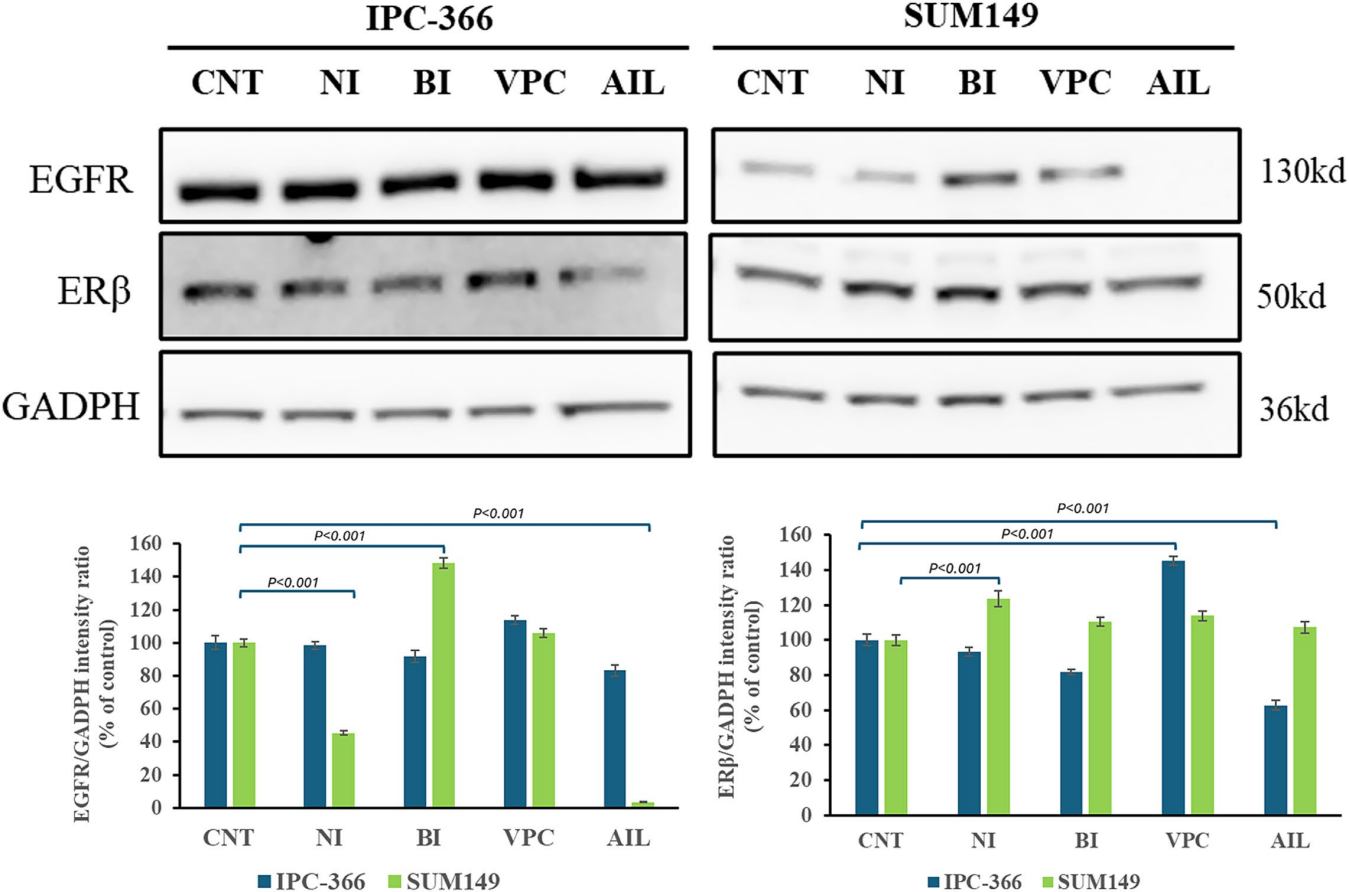
EGFR and ERβ expression in IPC-366 and SUM149 untreated and treated cells. GADPH expression was determined as loading control. Graphics of EGFR and ERβ expression quantification (from left to right). Bars represent protein intensity respect to GADPH expression and data was represented as percentage respect to CNT group.

Interfering in AR signaling with the administration of different AR antagonists resulted in differences in expression of other receptors related to AR signaling such as EGFR and ERβ (Figure 9). While EGFR expression was not altered in IPC-366 cells with all the treatments, in SUM149 cells EGFR expression decreased significantly (*p* < 0.05) with the administration of nilutamide and ailanthone, but significantly increased (*p* < 0.05) with bicalutamide treatment. Likewise, ERβ expression showed a significant increased (*p* < 0.05) with nilutamide treatment in SUM149 cells. IPC-366 cells, ERβ expression was significantly increased (*p* < 0.05) by VPC-13566 treatment, but significantly decreased (*p* < 0.05) by ailanthone treatment.

## Discussion

Endocrine therapies are known to have limited efficacy in TNBC patients, in part because of the lack of hormone receptors (ER and PR) expression. Thus, the use of these therapies is restricted to hormone receptor-positive breast tumors (4, 13, 20). However, several studies have shown that endocrine therapies, targeting steroid hormone production, showed beneficial results in *in vivo* and *in vitro* studies carried out using TNBC cell lines (26, 27, 31). Therefore, these treatments must exert their action through mechanisms that, currently, are scarcely studied. Despite not expressing the classical hormone receptors, TNBC tumors can express other hormonal receptors not contemplated on tumor diagnosis such as AR and ERβ. AR positivity has been reported between 20 and 50% in TNBC tumors (1, 5, 32) while ERβ positivity accounts for around 20–30% of these tumors (33, 34). The expression of these receptors in TNBC is beginning to gain relevance, since it may provide opportunities for the development of novel effective therapies (9, 13). Hence, the present study demonstrates that AR and the hormonal environment play an important role in TNBC progression and AR antagonists are effective against canine and human TNBC cell lines.

In this context, the role of AR results controversial in TNBC tumors; some studies suggest that AR acts as a driver of tumor progression and, other studies have shown that AR expression in TNBC is associated with lower histologic grade (5, 32). Therefore, there is a need of unraveled AR role in these tumors in order to propose it as an efficient therapeutic target.

For the hormone receptors to be activated, the presence of steroid hormones that bind to it is necessary, and the balance between androgens and estrogens is essential for vital functions (5, 35). It has been observed that tumor cells are capable of producing and secreting steroid hormones under both *in vitro* and *in vivo* conditions (29). Estrogen production promotes tumor cell proliferation and survival, while androgens are mainly involved in cell migration processes in TNBC cell lines (25). Therefore, steroid hormones exert an autocrine and paracrine effect on the tumor environment and can be considered strategic in tumor progression (30, 31).

Previously it was reported that the two TNBC cell lines used in this study (IPC-366 and SUM149) share biological and histopathological characteristics demonstrating that the canine cell line model can be a useful tool for comparative canine and human studies (24). Although these TNBC cell lines present multiple similarities, it has been reported that both cell lines were positive for AR and ERβ expression but differed on expression intensity, being this higher in IPC-366 cells than in SUM149 cells for both receptors (25, 26). Furthermore, this study revealed that IPC-366 cells secreted more estrogens, whereas SUM149 cells secreted more androgens, so the DHT/E2 ratio in IPC-366 was lower than in SUM149. These differences in receptor expression and steroid hormone secretion suggest variations in receptor signaling and also in the efficacy of endocrine therapies.

In fact, it is also demonstrated that AR expression is involved in cell proliferation, since when its expression is silenced under *in vitro* conditions, cell viability decreases in IPC-366 cells. *In vivo* results also confirmed that AR blockade can reduce tumor progression in SCID mice inoculated with IPC-366 siAR cells. In addition, the presence or absence of AR expression can modulate the hormonal environment of the tumor, which may be associated with this reduction in tumor progression. Mice inoculated with siAR cells presented a significantly higher blood DHT/E2 ratio than control mice, denoting an increase in circulating androgen levels that are associated with tumor progression reduction (31). Hence, the DHT/E2 ratio may provide information regarding tumor progression.

Also, the hormonal environment is indispensable for AR activation as *in vitro* results showed that an androgenic environment promotes proliferation through its activation. The AR can also be activated by androgen-independent pathways such as PI3K or MAPK signaling (6, 16, 33). However, this study demonstrates that in TNBC cells, AR is mainly activated by androgen-dependent pathways and can modulate these signaling pathways.

**Figure 10:**
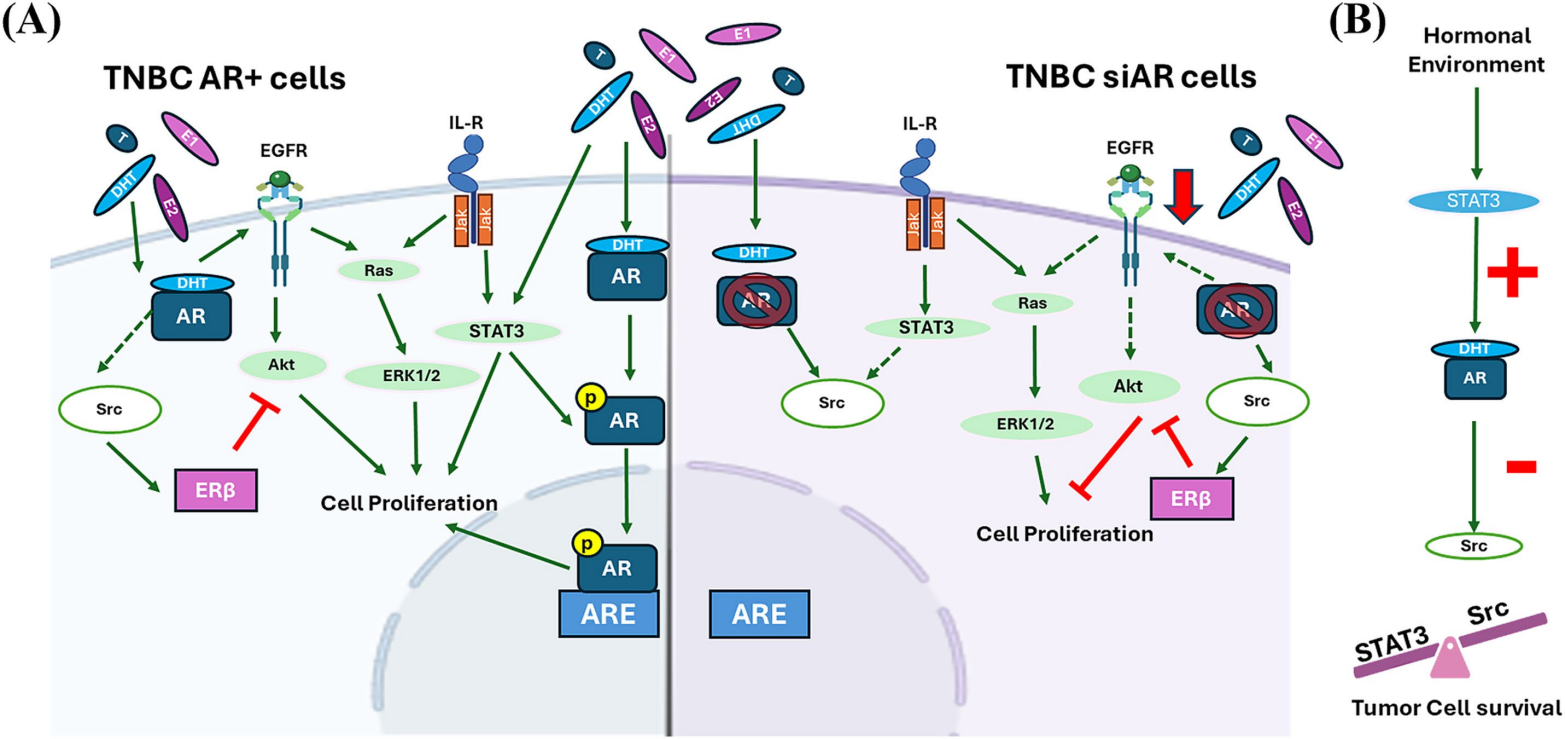
**(A)** AR signaling in TNBC AR positive (AR+) and negative (AR-) cells. In TNBC AR + cells, AR activation promotes cells proliferation directly or indirectly by upregulating EGFR activity, and consequently activating PI3K and MAPK signaling, and repressing Src activation. In TNBC AR- cells, Src is overexpressed and plays a pivotal role on cell survival. Src promotes cell proliferation by activating MAPK signaling, and also mediates the inhibition of cell proliferation activating ERβ by inhibiting PI3K signaling. Green continuous arrows denoted a positive regulation; green dashed arrows a negative regulation; and red lines an inhibition process. **(B)** Hormonal tumor environment also modulates STAT3 expression which regulates AR activation that exerts a negative regulation on Src expression. The balance between STAT3 and Src expression guarantees tumor cell survival.

Nevertheless, even in the absence of AR expression in TNBC cells, alterations in the hormonal microenvironment can influence the activation of these signaling pathways by altering the expression of molecules such as Ras or Akt and thus modulating cell proliferation (36). This may be because steroid hormones act through other receptors. Results showed that, in the absence of AR expression, there is an increase on ERβ expression that may block PI3K/Akt signaling and consequently inhibit cell proliferation, which is in accordance with other authors that demonstrated that ERβ interacts with PI3K/Akt signaling increasing PTEN expression and inducing apoptosis in TNBC cells, associating its expression with good prognosis (14, 37). Also, silencing AR expression resulted in a downregulation of EGFR and producing an antiproliferative effect in TNBC cells, as other authors demonstrate in ER + tumors (38). These results indicate that the non-genomic actions of AR are due to the AR-induced-activation of other receptors such as EGFR, which in turn activate MAPK and PI3K signaling pathways. Therefore, AR activation drives cell proliferation and survival by upregulating EGFR and downregulating ERβ activation (Figure 10A).

ERβ expression is also associated with Src expression, which acts as its co-activator (39). Src is a proto-oncogene that regulates cell proliferation, adhesion and modulates signaling pathways. In addition, this protein is recruited by steroid receptors (ER or AR) activating gene transcription (40), denoting Src expression can be modulated by AR (41). This can be demonstrated by comparing SUM149 and IPC-366 expressions. SUM149 presented low AR positivity but a high Src positivity and, on the contrary, IPC-366 cells showed high AR expression but low Src expression. Results also showed that Src expression increased in siAR cells compared to AR + cells, indicating a crosstalk between Src and AR. In these cells, Src expression can contribute to the activation of ERβ inhibiting cell proliferation and also can activate the MAPK signaling by increasing ERK1 expression and modulating cell proliferation (42). Therefore, in the absence of hormonal receptor expression, Src is responsible for modulating cell survival.

The hormonal tumor environment will also contribute to an AR-mediated response. Tumor cells, regardless of their AR expression, in a highly estrogenic environment tend to produce and secrete more androgens, whereas in a highly androgenic environment they secrete more estrogens. This homeostatic mechanism or adaptation of tumor cells to the surrounding environment may give them their ability to proliferate and survive. Indeed, the hormonal environment can interfere with signaling pathways. This study revealed that hormone administration (DHT or E2) to AR + cells decrease STAT3 expression, while in siAR cells this expression increased. These results suggest that STAT3 expression may be modulated by the hormonal environment. STAT3 is mainly activated by IL-6 receptor signaling and can also modulate AR activation in prostate cancer cells (43). IL6 is one of the most studied interleukins, and several studies show that androgens can suppress IL-6 secretion *in vitro* models, thereby influencing STAT3 expression (44). Therefore, the hormonal imbalance caused by the administration of steroid hormones may decrease interleukin secretion in vitro and interfere with STAT3 expression and, consequently, with AR activation. Other possible explanation may reside on Src expression. Results revealed a negative association between STAT3 and Src expression in IPC-366 cells; as STAT3 expression decreased, there was an increase in Src and vice versa. Some authors reported that Src inhibition may activate STAT3 for cell survival and proliferation (45). Taken together, these results suggest that hormonal signals can interfere with STAT3/AR activation and modulate Src expression. Thus, there must be a balance between Src and STAT3 expressions mediated by AR activation, which acts as a tumor resistance mechanism for cell survival (Figure 10B).

These findings establish that AR can be considered a therapeutic target as it plays a crucial role in the progression of TNBC. Therefore, the *in vitro* effect of different AR antagonists was evaluated in IPC-366 and SUM149 cell lines, with high and low AR positivity, respectively. For this purpose, two treatments that block the AR binding site (bicalutamide and nilutamide), and two compounds that block the AR transcription and its variants (VPC-13566 and ailanthone) were used (23).

The use of these AR antagonists showed a reduction in AR expression in IPC-366 cells with an increase in p-AR expression; whereas in SUM149 cells, these treatments were able to block AR and p-AR expressions. These treatments also affected androgen secretion. Although T levels were significantly decreased with all treatments and both cell lines, DHT levels were significantly reduced only in SUM149 which may be related to the blockade of AR expression. However, in IPC-366 DHT levels do not vary with treatments which may explain the partial reduction in AR expression found.

Previous studies reported that both cell lines were sensitive to AR antagonists such as flutamide or bicalutamide (25, 31). In accordance, results revealed that IPC-366 was more sensitive and reduced cell viability by a higher percentage with the administration of AR antagonists than SUM149 cells. However, these treatments were more effective in SUM149 in terms of cell migration than in IPC-366. It has been observed that androgen concentrations are related to migration processes, whereas estrogen levels have been related to proliferation processes (25). This is in agreement with the results obtained on hormone secretion, where it is observed that IPC-366 significantly reduced estrogen levels after the administration of the treatments, and SUM149 significantly reduced androgen levels. Therefore, these differences may be due to the fact that IPC-366 is more influenced by estrogens and therefore the changes are evidenced in terms of proliferation, while SUM149 is more influenced by androgens and therefore presents notable changes in terms of migration. These differences were also reflected in the DHT/E2 ratio after treatment administration. While the DHT/E2 ratio increases in IPC-366 cells that were more sensitive to AR antagonists, this ratio decreases in SUM149 cells that were less sensitive to the treatments. Therefore, the DHT/E2 ratio could be a good indicator of the efficacy of endocrine therapies in TNBC. However, this ratio still needs to be validated using a large number of samples and under various conditions.

In addition, in IPC-366 cells, no changes were found in the expression of molecules related to AR signaling, except for a decrease in ERK1/2 expression that may be associated with reduced cell proliferation (42). However, in SUM149 cells, AR antagonists produced an effect similar to that produced in IPC-366 siAR cells: in the absence of AR, there was an overexpression of Src that regulates ERβ and thus reduced cell proliferation. On the other hand, Src ensured cell survival by upregulating Ras and ERK2 expressions. The resemblance of the responses in IPC-366 siAR and treated SUM149 cells strengthens the canine model as a great research model for this disease in both species.

Expression of ARV7 was reduced in IPC-366 cells. Indeed, only ailanthone blocked ARV7 expression in IPC-366 cells. ARV7 is an AR RNA splicing alternative that results in a truncated AR protein that can be expressed in prostate and breast tumors. Although its expression is not well-studied in breast cancer models, ARV7 expression is associated with castration-resistant prostate cancer models and resistance to androgen deprivation therapies (17, 46, 47). In accordance, our results showed that ARV7 expression is maintained in IPC-366 siAR cells, but the hormonal environment can reduce its expression reducing cell proliferation and enhancing hormonal therapy efficacy. Thus, these results corroborate that ARV7 expression can be maintained in AR negative cells, but hormonal environment can modulate it. In fact, AR antagonists produced an increase of DHT/E2 in IPC-366 cells especially with ailanthone, which can be associated with the decrease of ARV7 expression found. Therefore, if ARV7 is involved in therapy resistance and the hormonal environment can modulate its expression, determining the hormonal microenvironment may help to prevent resistance to endocrine therapies.

From all the treatments analyzed, ailanthone presented more effectiveness in both cell lines in terms of cell proliferation and migration. Ailanthone is a natural compound extracted from the seeds of *Ailanthus altissima* that presented antitumoral activity but so far little is known about it. This small-molecule has been found to reduce AR full length and AR-variants protein expression resulting in a cell growth inhibition in prostate cancer (48). However, there is scarce information regarding the potential of this compound in breast cancer. Compared to other AR antagonists used, this compound appears to be more effective at blocking AR action by altering both its signaling and the hormonal environment. Results revealed that ailanthone was able to significantly reduce AR expression while also reducing Akt expression, thereby decreasing cell proliferation. In addition, ailanthone reduced the expression of ARV7 and Src, which may limit the activation of resistance mechanisms in these cells. Furthermore, ailanthone produces a large increase in androgen secretion leading to an increase in the DHT/E2 ratio suggesting its efficacy as a good endocrine therapy for TNBC tumors.

In summary, this study demonstrates that AR expression and the hormonal environment play a crucial role in tumor progression in TNBC, where an imbalance in hormonal homeostasis can trigger intracrine and paracrine signals that module AR function. This hormonal homeostasis will influence the expression of STAT3, which contribute to the activation of AR in TNBC cells. In turn, AR will modulate other receptors such as EGFR which, through PI3K and MAPK signaling, will promote cell proliferation processes. Furthermore, in the absence of AR, Src is overexpressed and ensures cell survival by activating the MAPK signaling and also regulates ERβ inhibiting PI3K pathway and cell proliferation. However, more specific mechanistic studies are needed in more TNBC models to confirm the AR-mediated function.

This study reveals that the presence of AR and ERβ in canine and human TNBC tumors may confer them sensitivity to endocrine therapies and that the DHT/E2 ratio proposed in this study could be an indicator of the efficacy of endocrine therapies in TNBC. Ailanthone has been shown to be an effective endocrine therapy in TNBC cell lines due to its dual function in blocking AR and Src and inhibiting cell proliferation and migration and blocking ARV7 expression truncating endocrine resistance.

## Conclusion

In conclusion, the expression of AR in canine and human TNBC promotes tumor progression by driving the expression of other proteins and receptors to ensure cell survival, and that novel compounds such as ailanthone can block AR activity and have been shown to be very effective in canine and human TNBC cells.

## Data Availability

The original contributions presented in the study are included in the article/Supplementary material, further inquiries can be directed to the corresponding author.

## Supplementary material

SUPPLEMENTARY FIGURE S1 Protein expression quantification (EGFR, STAT3, Src, Erβ, Akt, ERK1/2 and Ras) in IPC-366 control and siAR cells after DHT and E2 administration. **(A)** Bars represent siCNT and siAR protein intensity respect to GADPH expression and data was represented as percentage respect to control (CNT) group. **(B)** Graphs represent protein intensity respect to GADPH of siCNT cells (left) and siAR cells (right) with or without E2 and DHT administration. **(C)** Steroid hormone concentrations secretd by siCNT and siAR cells.
