# Supplementary figures and images for: Androgen receptor activation promotes tumor progression in canine and human triple negative breast cancer cell lines
Source: Front Vet Sci. 2025 Sep 23;12:1677830. doi: 10.3389/fvets.2025.1677830 (PMC12500424; doi:10.3389/fvets.2025.1677830)

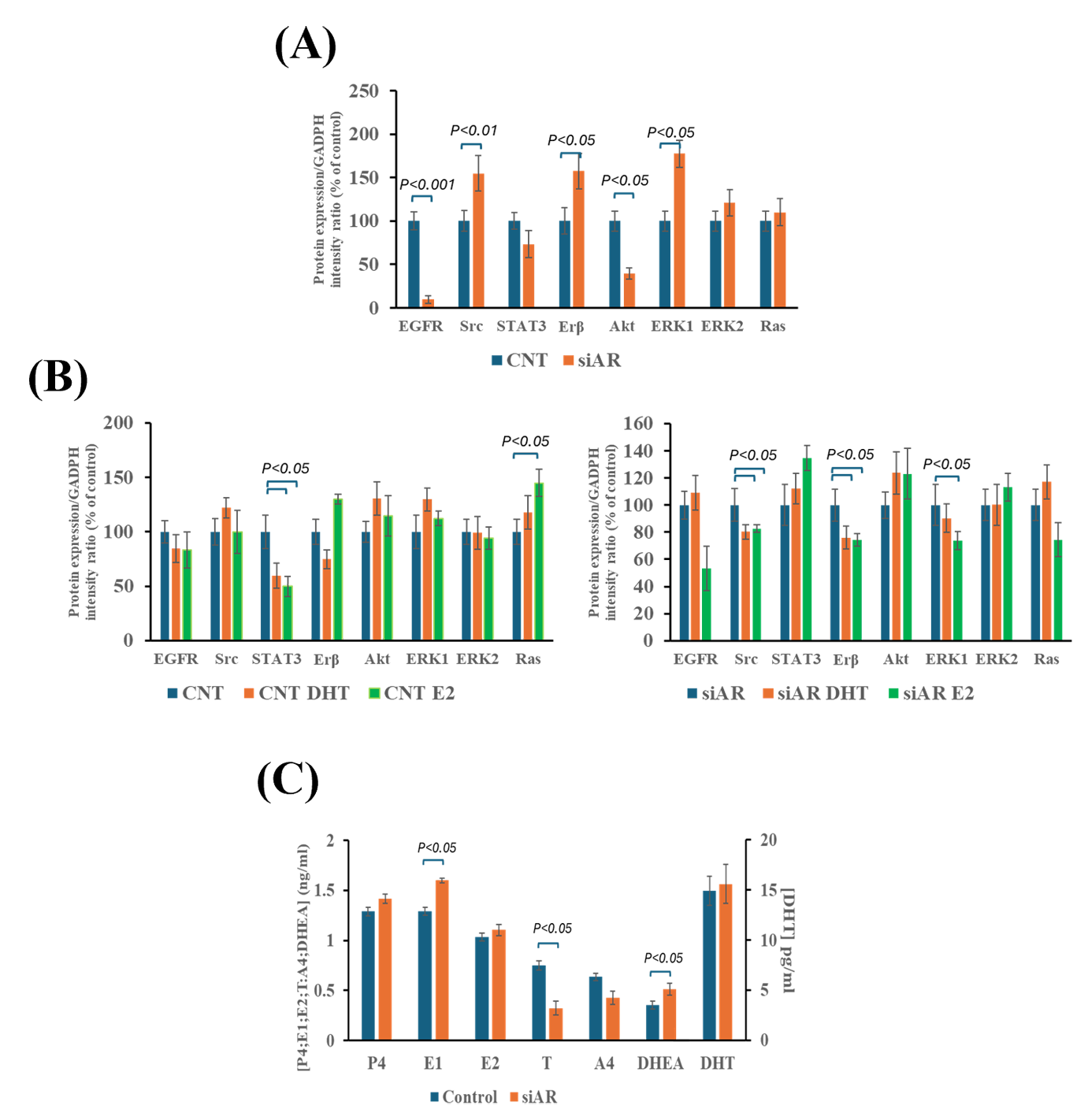

Supplement: SUPPLEMENTARY FIGURE S1 — Protein expression quantification (EGFR, STAT3, Src, Erβ, Akt, ERK1/2 and Ras) in IPC-366 control and siAR cells after DHT and E2 administration. (A) Bars represent siCNT and siAR protein intensity respect to GADPH expression and data was represented as percentage respect to control (CNT) group. (B) Graphs represent protein intensity respect to GADPH of siCNT cells (left) and siAR cells (right) with or without E2 and DHT administration. (C) Steroid hormone concentrations secretd by siCNT and siAR cells. [file Image_1.TIF]
